# Exploring Typologies of Immediacy Events in Psychodynamic Therapy: A Latent Class Analysis

**DOI:** 10.1002/jclp.23812

**Published:** 2025-05-30

**Authors:** Anna Mylona, Evrinomy Avdi, Julie Vaiopoulou

**Affiliations:** ^1^ School of Psychology, Faculty of Philosophy Aristotle University of Thessaloniki; ^2^ Department of Education University of Nicosia Nicosia Cyprus

**Keywords:** immediacy events, Latent Class Analysis, negotiation of the therapeutic relationship, psychodynamic therapy process

## Abstract

Immediacy is a therapeutic intervention that entails the explicit discussion between therapist and client about their relationship in the here‐and‐now. It is considered a potentially powerful intervention that can facilitate relational processing, especially in psychodynamic psychotherapies. Τhis study aims to explore the use of immediacy in psychodynamic psychotherapy through a case‐series analysis. Videos of 139 sessions, drawn from 7 psychotherapies with 2 therapists, were coded in terms of the type of immediacy used and its immediate effects, as reflected in the client's response. A total of 121 immediacy events were identified in 57 sessions, occupying 8% of the total therapy time; the majority were initiated by the therapist. A Latent Class Analysis was conducted to explore where there exist clusters of immediacy events that share patterns of immediacy type and effect. Two distinct clusters of immediacy events were identified. The most common group, which we termed ‘limited engagement in immediacy’, was characterised by a primarily insight‐oriented agenda on the therapist's part, smooth collaboration during the immediacy event, and limited engagement on the client's part. The second cluster, termed ‘mutual engagement in immediacy,’ reflected more complex processes characterised by conflict, with the therapist using a range of interventions (from alliance‐building to insight‐oriented interventions), evidence of disruption in the therapist‐client collaboration, and the client showing increased engagement and reporting improvement. The findings are discussed drawing upon the literature on the processes of repairing ruptures in the therapeutic alliance and clinical implications regarding the use of immediacy.

## 1 Introduction

There is ample evidence that the therapeutic relationship, and more specifically the quality of the therapeutic or working alliance, are important common factors in therapy process and robust predictors of treatment outcome, regardless of therapeutic modality (Flückiger et al. 2018; Horvath et al. 2011). Notwithstanding this, it is generally accepted that negative relational processes, such as ruptures in the therapeutic alliance, are inevitable in therapy and recent research suggests that repairing ruptures may constitute a mechanism of change (Norcross and Lambert 2018). One way in which alliance ruptures can be repaired is through processing the therapeutic relationship in the here‐and‐now (Safran and Muran 2000). However, the research evidence on how such working through contributes to the process and outcome of therapy remains unclear (Hill and Knox 2009).

Recently, immediacy has attracted attention as one way of operationalizing relational processing. Hill (2009) introduced the term ‘therapist immediacy’, which she defined in terms of the therapist disclosing their immediate feelings toward the client and their relationship in the here‐and‐now of the session. Kuutmann and Hilsenroth (2012) extended this definition to that of ‘therapeutic immediacy’ whereby both therapist and client are considered to contribute to immediacy discussions, thus approaching immediacy as an interactive, dyadic process. In their formulation, therapeutic immediacy entails “any discussion within the therapy session about the relationship between therapist and patient and any processing of what occurs in the here‐and‐now patient‐therapist interaction” (Kuutmann and Hilsenroth 2012, p.188). Metacommunication is another term that is often used interchangeably with that of immediacy (Kiesler 1988). Metacommunication concerns explicit communication about the therapeutic communication, with the therapists sharing their immediate experience of their interaction with the client and explicitly commenting upon the client's verbal and non‐verbal behaviours that contribute to specific in‐session transactions (Safran and Muran 2000; Wagner and Safran 2010). As such, metacommunication has been described as the beginning of a process of “disembedding” oneself from an enactment ‐i.e., reflecting the contribution of both therapist and client in forming specific patterns of interaction‐ that leads to the client developing self‐assertion and reflection about the here‐and‐now (Safran and Muran 2000).

Recent empirical evidence on immediacy suggested it as a potentially important intervention for dealing with problems as they arise in the therapeutic relationship and can help clients to open up, be more immediate and gain insight; on the other hand, it has also been described as a ‘risky’ intervention potentially associated with negative outcomes (Hill et al. 2018). Next, we provide an overview of the emerging research on immediacy before turning to the study.

### 1.1 Empirical Research on Immediacy

The first studies on immediacy were case studies on interpersonal and psychodynamic psychotherapy that explored its clinical utility by investigating its use and its effects (Hill et al. 2008; Kasper et al. 2008; Mayotte‐Blum et al. 2012). A sophisticated consensual approach, named Consensual Qualitative Research (Jackson et al. 2012), has lately been used for research on immediacy and tends to be considered methodology of choice in the field. In these studies, trained judges identify immediacy events, i.e., periods in the session where the conversation shifts to the therapeutic relationship, and subsequently rate the characteristics and quality of these events, through consensus. These studies suggest that immediacy is generally a helpful intervention that may facilitate client emotional expression, awareness and, insight, may provide corrective relational experiences, and may reduce clients' defenses, help negotiate problems in the therapeutic relationship and model, in this way, the resolution of interpersonal problems outside therapy (e.g., Hill et al. 2008; Hill et al. 2014; Mayotte‐Blum et al. 2012).

A meta‐analysis of studies on immediacy indicates that its prevalence ranges from 5% to 38% of total therapy time across cases, with the therapists' theoretical orientation (psychodynamic or interpersonal) moderating its use (12–38%) (Hill et al. 2018). Immediacy has been shown to be more extensively used by experienced therapists (Hill et al. 2008; Kasper et al. 2008; Mayotte‐Blum et al. 2012; 12%, 34%, and 38%, respectively) when compared to doctoral student therapists (5% of total therapy time, ranging from 0.5% to 14% of total therapy time through the cases) (Hill et al. 2014). Furthermore, studies have shown that immediacy events were most often initiated by the therapist, ranging from 50% to 100% of cases (Hill et al. 2008; Kasper et al. 2008; Mayotte‐Blum et al. 2012).

Six general immediacy types have been reported in the latest meta‐analytic review on immediacy (Hill et al. 2018). Immediacy types refer to specific strategies employed by the therapist in the context of immediacy discussions and include the following: (i) *probes and inquiries about the relationship*, where the therapist invites the client to share feelings about the therapeutic relationship in the moment (ii) *therapist's statement of their reactions to the client*, where the therapist shares their reactions and feelings toward the client in the here‐and‐now (iii) *making the covert overt*, where the therapist comments on the client's covert, unexpressed reactions, feelings or interpersonal patterns, thus inviting the client to explore these, (iv) *drawing parallels with outside relationships*, where the therapist wonders whether the client has similar reactions and feelings toward them as they have toward important others in their life (v) *acknowledgement of a breach in the relationship*, where the therapist explicitly recognizes the ruptured communication *and* (vi) *attempts at repairing a rupture*, where the therapists indents to secure alliance, through validating the client's feelings and defences, acknowledging their contribution for mistakes, clarifying misunderstandings, negotiating and providing a rationale for treatment tasks. Research to date has shown that the most commonly used immediacy types are the exploration of covert feelings, drawing parallels with outside relationships, and to a lesser extent, the acknowledgement of a breach in the relationship and therapists' statement of their emotions (Hill et al. 2014). These immediacy strategies resemble the rupture repair strategies proposed by Safran and Muran (2000) for processing alliance ruptures in the here‐and‐now.

The effects of immediacy on the therapy process have been assessed in primarily two ways, namely, coding the immediate outcome of immediacy as reflected in the clients' response to the immediacy intervention and by clients' retrospective reports of their experience of immediacy and their views on its role in the process of therapy (Hill et al. 2014). In these studies, the use of immediacy has been primarily associated with helpful processes, such as enhanced therapy relationship, the provision of corrective emotional experience, the client opening up and displaying increased emotional expression, engaging in immediacy talk, and showing improved interpersonal functioning (Hill et al. 2018). However, some less helpful effects have also been reported, such as an impaired therapeutic climate and the client's inhibition, puzzlement, and discomfort in response to the intervention (Hill et al. 2014; Kasper et al. 2008). The effects of immediacy use in terms of session‐level outcome (clients' perception of session quality and alliance) and treatment outcome have been investigated through mixed‐method case‐series. More specifically, Hill et al. (2014), in a 16 case‐series study, found no association between the amount and duration of immediacy events with clients' ratings of session quality and changes in interpersonal functioning. Shafran et al. (2016) investigated within‐client effects of immediacy use in the same 16 cases and found that a higher frequency of immediacy use in a session was related to higher client ratings of session quality. Interestingly, a higher frequency of immediacy was also found to be associated with worse client‐rated alliance early in treatment and better client‐rated alliance later in treatment, indicating that the timing of immediacy use is important.

Given the mixed findings regarding the effects of immediacy on therapy process, the question arises as to what are the characteristics of effective immediacy interventions. In the few studies examining this question to date, the effectiveness of immediacy interventions has been studied in relation to the degree of the clients' involvement in immediacy and the quality of immediacy effects, as rated by trained judges. A recent study suggested that high‐quality immediacy events, as rated by judges, were associated with higher client involvement; as such effective immediacy interventions were considered to keep clients involved in reflective and productive immediacy conversations (Kuprian et al. 2022). Preliminary results suggest that increased client involvement in immediacy was associated with the therapist's inquiries about the client's immediate feelings and the therapist disclosing their own immediate feelings; drawing parallels with extra‐therapy relationships was not found to be associated with increased client involvement in immediacy and the results were mixed regarding the therapist's use of feedback about the client's in‐session behaviour, e.g., commenting on client's avoidance (Kasper et al. 2008; Kuprian et al. 2022).

In another set of studies, the quality of immediacy interventions was examined by Hill et al. (2008), who distinguished between supportive and challenging immediacy interventions. Supportive immediacy interventions entailed the maintenance of collaboration through reinforcement and acknowledgement of the client's in‐session behaviour and through inquiries about their reactions to therapy, and these were found to be associated with positive immediacy effects (such as increased client emotional expression and involvement in immediacy, negotiation of therapeutic relationship) and with better treatment outcome. Challenging immediacy interventions aimed at reducing the client's defences through drawing parallels with outside relationships and encouraging clients to express their immediate feelings and reactions to the therapist, and these were found to be associated with both positive and negative effects, limited client involvement in immediacy, and mixed findings with regard to treatment outcome.

In sum, immediacy is considered an impactful therapeutic skill; however, the mechanisms through which it contributes to the therapeutic alliance and therapy outcome are far from known, as are the characteristics of effective immediacy interventions. The investigation of specific immediacy interventions (e.g., supportive, interpretive, challenging, self‐involving, providing feedback etc.) and their association with immediate, in‐session effects merits further investigation and could inform clinicians concerning the effective delivery of immediacy interventions.

### 1.2 Present Study

In psychodynamic practice, the processing of the therapeutic relationship and the promotion of relational awareness regarding the dyad's enacted maladaptive interpersonal patterns in the here‐and‐now are assumed key to therapeutic change; this processing is often promoted through transference work and transference interpretations (Freud 1912; Strupp and Binder 1984). In this study, our overall aim is to explore the therapeutic practice of immediacy in psychodynamic therapy and to formulate hypotheses regarding the characteristics of effective immediacy interventions. More specifically, we aim to investigate the prevalence and characteristics of immediacy events as they emerge in routine clinical practice, namely the types most commonly used and their immediate effects on the interaction. Additionally, we aim to explore some preliminary associations of immediacy types and immediate effects through the identified immediacy events.

For this purpose, we conducted a systematic observation of immediacy events in 139 video‐recorded sessions of face‐to‐face psychodynamic therapy delivered by experienced clinicians in an outpatient service. Trained judges consensually identified immediacy events and categorized them in terms of type and immediate effects.

## 2 Methods

### 2.1 Data Set

The material for this study was collected in the context of a broader research project that aims to investigate therapeutic interaction in naturalistic settings (Avdi and Seikkula 2019; Seikkula et al. 2015). The data were collected over a 3‐year period in a community mental health centre providing open‐ended individual psychodynamic therapy in Greece. The research material consists of 139 video‐recorded sessions of individual psychodynamic therapy drawn from seven psychotherapies conducted by two therapists. The number of sessions per case ranged from four to 52 (*M* = 20, SD = 17).

### 2.2 Participants

#### 2.2.1 Clients

Seven clients participated in this study (five women, two men); all clients were white European, and their ages ranged from 22 to 37 years (*M* = 30, SD = 5.92). No formal diagnoses were determined, and the presenting problems included depression, anxiety, and interpersonal difficulties.

#### 2.2.2 Therapists

Two experienced female therapists, one a psychiatrist and psychoanalyst and the other a clinical psychologist, each with over 25 years of clinical experience, participated in the study. Therapist 1 treated five clients (two men and three women) over 76 sessions, and Therapist 2 treated two clients (both women), conducting 63 sessions overall. Both therapists and clients were unaware of the specific aims of this study at the time of data collection.

### 2.3 Procedures for Data Collection

Given the naturalistic design of the study, no specific inclusion criteria were used for participants' recruitment. Prospective clients were informed about the study by a member of staff at the intake meeting, and if interested, they were fully informed about the project by a graduate researcher. During the data collection period (2016–2018), seven clients agreed to participate in the study. Our initial intention regarding the data collected per case was to video‐record the first year of therapy. Ethical approval for the study was provided by the Scientific Committee of the mental health centre, and written informed consent was obtained from all participants.

#### 2.3.1 Treatment

The sessions were weekly, face‐to‐face, and lasted 50 min. The therapy was open‐ended, non‐manualized psychodynamic therapy. The therapists worked in their usual clinical style, and all sessions were video‐recorded by a web camera, unobtrusively positioned in the consulting room.

### 2.4 Procedures for Coding Immediacy Events

The rating team consisted of two coders, a doctoral researcher and a graduate student. Due to the lack of an established coding system for immediacy, we devised a systematic way of coding immediacy events inspired by the Consensual Qualitative Research paradigm (Hill & Knox 2021; Jackson et al. 2012). Immediacy events were coded in terms of (a) immediacy type(s) employed and (b) its immediate effects in terms of the client's response, drawing upon existing literature (Hill et al. 2018). The coders initially familiarized themselves with the theoretical and empirical literature on immediacy. They reviewed the criteria for immediacy identification and previously used categories of immediacy types and immediate effects and used these in their coding procedure.

In coding the sessions, the following principles were followed. Initially, Immediacy Events (IEs), the unit of analysis, were determined in each session. IEs are defined as periods in a session during which the conversation focuses explicitly on the therapeutic relationship; both therapist and client should be involved at least minimally in the discussion, and the discussion should address the therapeutic relationship in more than a social chitchat manner or practical discussion about the sessions (Hill and Knox 2009). The starting point of each IE was defined as the point in the session when the discussion focused explicitly on the therapeutic relationship and the end‐point when there was a shift to another topic.

Each coder watched videos of the sessions independently and identified IEs; then, the coders met, shared their respective codings and discussed any disagreements until consensus was reached. Then, they determined each IE's temporal delimitation and duration. This process was followed for the whole data set (139 sessions). No inter‐rater reliability score was calculated, as all codings used in the analysis were identified through consensus.

After identifying all IEs, the following frequencies were calculated: (i) the number of sessions with at least one IE, (ii) the total number of IEs in the data set, (iii) the total and average duration of IEs (in seconds) and (iv) the proportion of therapy time spent in immediacy discussions. The identified IEs were transcribed verbatim by two graduate students, removing any identifiable information, and checked for accuracy by the coding team.

Next, the coders rated all identified IEs together through consensus meetings. For each IE, the coders read the transcript (and, if necessary, watched the session video again) and determined the following: (a) whether each IE was preceded by another in‐session IE b) who initiated the IE (client or therapist), c) the immediacy type employed, and d) the immediate effects observed.

Immediacy types and effects of the IEs were categorized drawing upon existing literature (Hill et al. 2018). More specifically, six categories of immediacy types and thirteen categories of immediate effects were used for coding (Table 1). Immediate effects were coded depending on the clients' responses within the IEs and ranged from helpful (e.g., client's functioning improvement, client opening up, insight, client engaging in immediacy, relationship enhancement) to hindering effects (e.g., client's exploration inhibited, therapeutic collaboration impairment). It should be noted that each IE could be assigned to more than one immediacy type and effect, as it is typical in this kind of research field and approach of coding immediacy. It is worth noting that only the presence of a type and/or immediate effect was identified and not its significance for the interaction.

**Table 1:** Prevalence of immediacy types and immediate effects.

	Frequency							
	Case 01	Case 02	Case 03	Case 04	Case 05	Case 06	Case 07	Total
**Type of immediacy**								
1. Probes and inquiries about the relationship/Relational inquires	11	2	1	10	8	2	14	48
2. Therapist's statement of her reactions to the client	7	0	1	2	1	0	4	15
3. Making the covert overt/Explicit exploration of clients' implicit feelings	21	2	3	14	8	6	24	78
4. Drawing parallels with outside relationships/Drawing interpersonal parallels	15	0	1	9	8	2	7	42
5. Acknowledging a rupture in the relationship	3	0	0	0	0	2	0	5
6. Attempts to repair ruptures/Immediate repair	13	0	0	11	0	3	12	39
**Type of immediacy effect**								
1. Client's report of functionality improvement	0	1	0	1	2	1	3	8
2. Client's opening up and exploring experienced feelings	20	1	4	8	5	1	10	50
3. Client's insight gain	6	0	0	5	1	2	6	20
4. Client felt understood, normalized, and reassured	0	0	0	0	0	0	0	0
5. Client's use of immediacy/Client engagement in immediacy	19	1	1	8	4	3	19	55
6. Overall helpful for the client	0	0	3	3	1	3	2	12
7. Enhanced therapeutic relationship	5	0	1	3	6	1	1	17
8. Impaired therapeutic relationship/Impaired collaboration	5	0	1	9	0	1	5	21
9. Client's negative feelings and reactions	0	0	0	1	0	0	0	1
10. Client's openness/Exploration/Insight was inhibited	2	0	0	1	3	3	3	12
11. Overall not helpful for the client	0	0	0	1	1	0	1	3
12. Negative effects for the therapist	0	0	0	0	0	0	0	0
13. Overall neutral reactions/No changes for the client	1	2	0	0	8	0	1	12

*Note:* Based on 121 IEs across all cases. Categories were not mutually exclusive. Each category was counted once per event.

### 2.5 Latent Class Analysis

Besides the descriptive analysis conducted regarding the use of immediacy in the sample, we conducted a preliminary exploration of possible typologies of IEs, by which we mean IEs with shared characteristics (namely immediacy effects and types) providing thus preliminary associations of types and effects. The empirical data were strictly categorical, recorded as 1 or 0, indicating whether a particular immediacy type or a particular effect was observed. Since the aim was to examine the association of such categories in each IE, the statistical analysis of Latent Class Analysis (LCA) was chosen to detect existing clusters of IEs that share similar patterns of concurring categories.

LCA is an advanced method of analysis that uses Bayesian statistics to explore associations between categorical multivariate data and classify participants into qualitatively distinct groups, the latent classes, based on a set of response patterns. However, in the present paper, LCA was not used as a psychometric method but as a model‐based classification procedure handling the categorical data derived from the sessions' coding. LCA has lately numerous applications in clinical psychology (e.g., Cavanaugh et al. 2012; Georgaca et al. 2022, 2023; Klonsky and Olino 2008; Petersen et al. 2019; Tyndall et al. 2018), suggesting that it is a suitable analysis method for research in clinical psychology.

In LCA, the classification is based on a set of conditional probabilities (CP), which refer to the probabilities of observing a particular pattern of observations given the specific cluster (Clogg 1995; Dayton 1998). Based on several criteria, such as the classification error, the number of parameters, likelihood ratio statistic (L2), Bayesian Information Criterion (BIC), Akaike's Information Criterion (AIC), degrees of freedom, and bootstrapped *p*‐value, the researcher decides on the cluster‐model that best fits the data (Bakk et al. 2013; Magidson and Vermunt 2001). An advantage of LCA is that external factors could be used as independent or dependent variables and be associated with the ensued clusters. Given the limited research in the field, the analytic approach was exploratory, without specific hypotheses concerning the ensuing latent classes. The latent classes were determined inductively from the classification procedure based on the input criteria. LCA was performed with *Latent Gold5*.*1* software.

## 3 Results

### 3.1 Descriptive Analysis of Immediacy Interventions

Of the 139 sessions, 57 (41%) included at least one IE. One hundred and twenty‐one IEs were identified in total, with the average number of IEs per session being 2 (SD = 1.4). The mean duration of IEs was 250 s, although great variation was observed between events (SD = 388, *Median* = 114 s, ranging from 10 to 2949 s); 7.75% of the total treatment time was spent in immediacy, again with great variation between cases (ranging from 1 to 25.5% of the total treatment time per case), and therapists initiated 79% of the IEs.

Immediacy events tended to occur in clusters, both within sessions (whereby, 63 IEs (52%) were preceded by another IE in the same session) and between sessions (whereby, 40 sessions with IEs (70%) occurred successively). Interestingly, client nonattendance often preceded sessions with IEs (25 non‐shows (44%) preceded session with IEs). In terms of content, immediacy discussions tended to focus on the clients' disengagement and/or ambivalence regarding the therapeutic process.

The frequencies of immediacy types and immediate effects observed overall and per case are displayed in Table 1.

### 3.2 LCA: Analysis and Results

The unit of analysis was the Immediacy Event, the IE. All categories of immediacy types and effects (Table 1) were included in the initial analysis, and only those with statistically significant differences remained in the model presented. The input for analysis included: a. (client's) ‘*Report of functionality improvement*,*’* b. (client) ‘*Engagement in immediacy*,*’* and c. ‘*Impaired collaboration*,*’*. Specific therapist's immediacy types, i.e., i. ‘*Relational inquiries’*, ii. ‘*Explicit exploration of client's implicit feelings,’* iii. ‘*Drawing interpersonal parallels*,*’* and iv. ‘*Immediate repair’*, were included as covariates allowing us to understand if the classification differs by them.

Table 2 shows the results from the LCA. As observed, compared to the alternative models, the two‐cluster solution is the best parsimonious model in terms of BIC and AIC values and classification error. In Table 3, the statistical significance of the three input variables (immediate effects) is depicted (the associations of the clusters with covariates are shown in Table 4). All three differ significantly between the clusters.

**Table 2:** Results of LCA.

	LL	BIC (LL)	AIC (LL)	AIC3 (LL)	Npar	L²	df	*p*‐value	Class. Err.
1‐Cluster	−168.67	351.73	343.34	346.34	3	126.54	102		0
**2‐Cluster**	**−126.96**	**306.68**	**275.93**	**286.93**	**11**	**43.13**	**94**	**0.063**	**0.0459**
3‐Cluster	−122.31	335.74	282.62	301.62	19	33.82	86	0.081	0.0487
4‐Cluster	−119.89	369.26	293.77	320.77	27	28.97	78	0.1311	0.1196
5‐Cluster	−116.83	401.51	303.66	33.66	35	22.86	70	0.1134	0.1269

*Note:* Bold font denotes the selected cluster.

**Table 3:** The resulting two‐cluster‐model.

		Cluster1	S.E.	*z*‐value	Cluster2	S.E.	*z*‐value	Wald	*p*‐value	R²
(client's) Report of functionality improvement	Not observed	0.4287	0.2167	1.98	−0.4287	0.2167	−1.98	3.91	0.0480	0.0442
	Observed	−0,4287	0.2167	−1.98	0.4287	0.2167	1.98			
(client's) Engagement in immediacy	Not observed	0.8705	0.1765	4.93	−0.8705	0.1765	−4.93	24.32	0.0000	0.427
	Observed	−0.8705	0.1765	−4.93	0.8705	0.1765	4.93			
Impaired collaboration	Not observed	0.901	0.2543	3.54	−0.901	0.2543	−3.54	12.56	0.0004	0.2986
	Observed	−0.901	0.2543	−3.54	0.901	0.2543	3.54			

**Table 4:** The association of the two clusters with the immediacy types.

Covariates		Cluster1	S.E.	*z*‐value	Cluster2	S.E.	*z*‐value	Wald	*p*‐value
Relational inquires	Not observed	1.0085	0.4501	2.24	−1.0085	0.45	−2.24	5.02	0.025
	Observed	−1.0085	0.4501	−2.24	1.0085	0.45	2.24		
Explicit exploration of the client's implicit feelings	Not observed	1.5552	0.634	2.45	−1.5552	0.634	−2.45	6.02	0.014
	Observed	−1.5552	0.634	−2.45	1.5552	0.634	2.45		
Drawing interpersonal parallels	Not observed	1,2509	0.5045	2.48	−1.2509	0.505	−2.48	6.15	0.013
	Observed	−1.2509	0.5045	−2.48	1.2509	0.505	2.48		
Immediate repair	Not observed	1.2589	0.4436	2.84	−1.2589	0.444	−2.84	8.05	0.0046
	Observed	−1.2589	0.4436	−2.84	1.2589	0.444	2.84		

**Figure 1:**
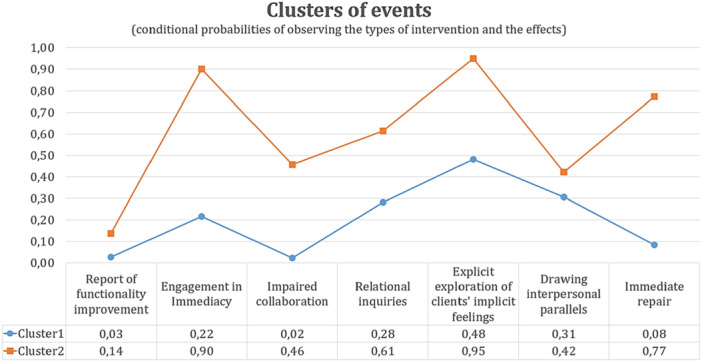
*The conditional probabilities in each input item regarding the event Note:* (Value of 1 = Observed) for the two Clusters. All differences are statistically significant.

Figure 1 shows the CP in each input item for the two clusters. Cluster 1 corresponds to 65.25% of the IEs and includes cases where, based on CPs, the three effects, i.e., (clients’) ‘report of functionality improvement,’ (client's) ‘engagement in immediacy,’ and ‘impaired collaboration,’ do not concur. Cluster 2 corresponds to 34.75% of the IEs and includes cases where the three effects co‐occur or, more precisely, have a large probability of co‐occurring.

The most notable difference in CPs between the clusters is found in clients' ‘engagement in immediacy’ (Cluster 1 − 22%, Cluster 2 − 90%). Regarding the other effects, a large difference is observed in ‘impaired collaboration’ (Cluster 1 − 2%, Cluster 2 − 46%). The relatively low CP in (client's) ‘report of functionality improvement’ for both clusters is due to the small number of cases; however, the differences between the two clusters are statistically significant, as depicted in Table 3, which shows the parameters of the two–cluster model.

The two clusters also differ in the immediacy types employed by the therapist, which were implemented as covariates in the model. The immediacy type with the higher CP of occurring in both clusters was the ‘explicit exploration of the client's implicit feelings’ type (Cluster 1 − 48%, Cluster 2 − 95%), followed by the ‘relational inquiries’ type (Cluster 1 – 28%, Cluster 2 – 61%). The most considerable difference in the CPs is observed in the ‘immediate repair type’ (Cluster 1 – 8%, Cluster 2 – 77%), and the smallest (although statistically significant) difference is seen in the CP of the ‘drawing interpersonal parallels’ type (Cluster 1 − 31%, Cluster 2 − 42%).

**Figure 2:**
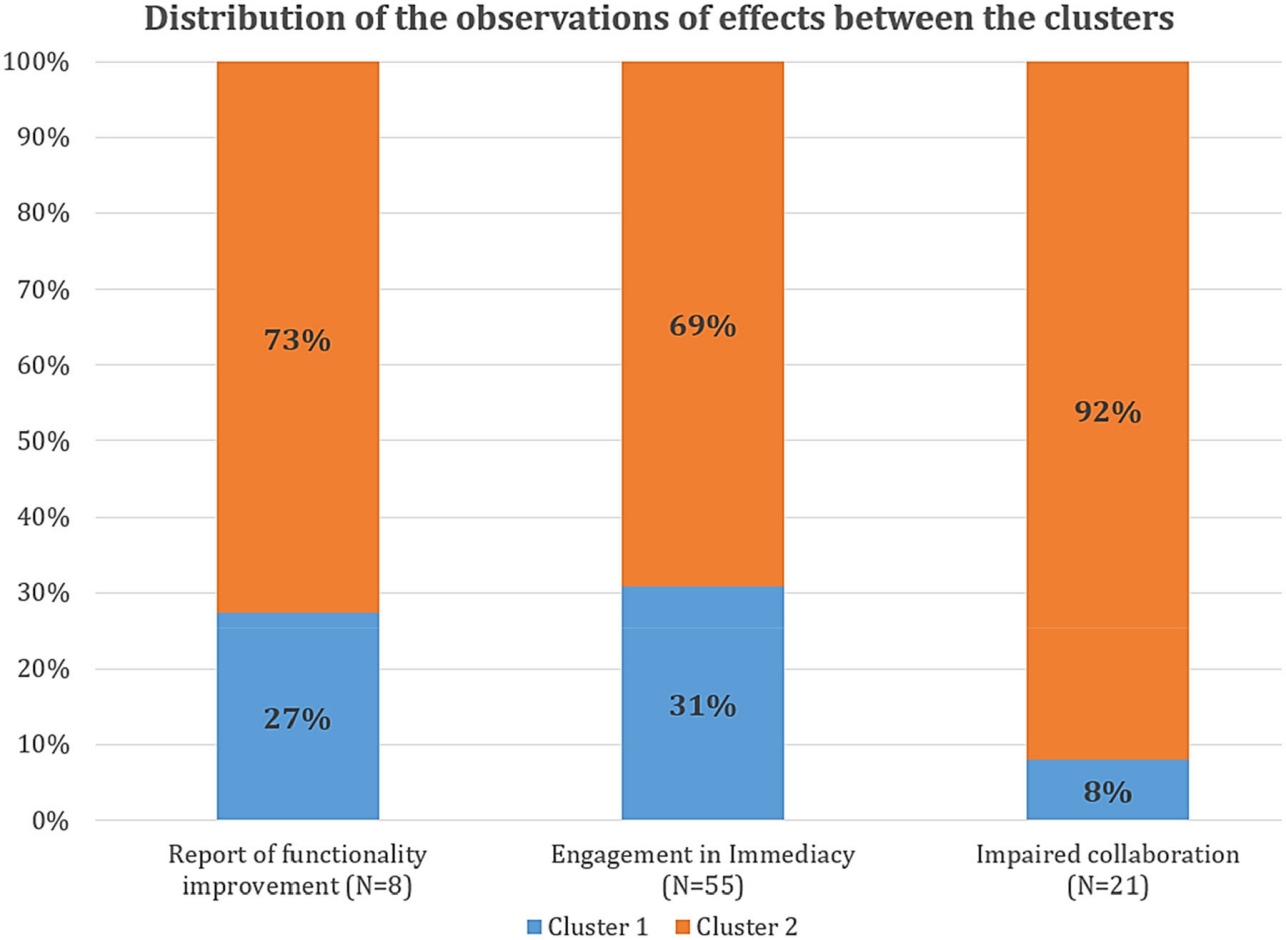
*Distribution of the observations of effects between the two clusters*. *Note:* Percentages of the total observations of each effect.

Figure 2 depicts the distribution of immediate effects between the two clusters. It is evident that Cluster 2 includes the cases where all the effects are most likely to be observed. Specifically, it includes 73% (out of 8) observations of clients reporting that their functioning had improved, 69% (out of 55) observations of clients getting engaged in the immediacy process, and 92% (out of 21) observations of impaired collaboration.

As stated previously, the LCA was applied with four covariates: ‘relational inquires,’ ‘explicit exploration of the client's implicit feelings,’ ‘drawing interpersonal parallels,’ and ‘immediate repair,’ which were associated with cluster membership. They all had a statistically significant association with the clusters, as depicted in Table 4. More specifically, Cluster 2 is positively associated with the occurrence of ‘relational inquiries’ (*b* = 1.0085, *z‐*value = 2.24, *Wald* = 5.02, *p* < 0.05), ‘explicit exploration of client's implicit feelings’ (*b* = 1,5552, *z*‐value = 2,45, *Wald* = 6,02, *p* < 0,05), ‘drawing interpersonal parallels’ (*b* = 1.2509, *z*‐value = 2.48, *Wald* = 6.15, *p* < 0.05), and ‘immediate repair’ (*b* = 1.2589, *z*‐value = 2.84, *Wald* = 8.05, *p* < 0.01). Since all variables were binary, the coefficients of associations in Cluster 1 were equal but with a negative sign. The observation of the immediacy types is positively associated with Cluster 2, where the CP of the observation of the immediacy effects is significantly higher.

## 4 Discussion

This study examined the use of immediacy, which we consider a key intervention to promote relational processing, which in turn lies at the heart of psychodynamic therapeutic action. It provides a preliminary model of immediacy use through the application of a Latent Class Analysis (LCA), which identified clusters of immediacy events (IEs) based on the observed immediate effects and their association with immediacy types. These initial findings contribute to the formulation of clinically‐relevant hypotheses regarding the use of immediacy, which need to be explored in studies with larger samples.

Immediacy was an intervention that was used relatively infrequently, occupying approximately 8% of total therapy time across all cases. It is worth mentioning that great variation was observed in the use of immediacy between cases, ranging from 1.02% to 25.5% of the total therapy time per case. This prevalence is higher than that reported in other case‐series studies, which report immediacy use ranging from 0.5% to 14% of total therapy time (Hill et al. 2014). This could be interpreted as reflecting the therapists' psychodynamic orientation in this study (Hill et al. 2018).

In addition, in this data set IEs tended to occur in clusters ‐both within and between sessions‐ and sessions with IEs tended to be preceded by client nonattendance. These observations have not been reported in previous studies on immediacy but are in line with studies that examine the emergence of alliance ruptures over the course of treatment and identified phases in therapy characterized by ‘alliance struggles’ (Schenk et al. 2019). As such, immediacy processes can be seen as a way of negotiating and processing alliance ruptures and client disengagement from the therapeutic process (Safran and Muran 2000).

The most frequently employed immediacy types in this data set included the therapist employing the ‘explicit exploration of client's implicit feelings,’ ‘relational inquires,’ ‘drawing interpersonal parallels,’ ‘immediate repair,’ and to a lesser degree, the therapist's ‘statement of immediate feelings and reactions toward client’ and ‘acknowledgment of ruptures.’ Similar frequencies of immediacy types have been reported in other case‐series studies of psychodynamic therapy (Hill et al. 2014). Overall, the range of immediacy practices employed can be understood as reflecting a psychodynamic agenda that aims, on the one hand, to foster relational insight, through promoting awareness and exploration of the clients' implicit, unconscious feelings and enacted maladaptive interpersonal patterns in the here‐and‐now, while also at safeguarding the therapeutic alliance, through providing acknowledgement and validation of clients' feelings (Gabbard 2014; Safran and Muran 2000).

From the application of LCA, two distinct clusters of IEs were identified, with the first pertaining 65% and the second 35% of the sample. The first, more common, cluster, ‘limited engagement in immediacy,’ was characterised by smooth, presumably attuned immediacy processes as reflected in a positive therapeutic climate, with relatively limited engagement in reflective discussions about the here‐and‐now, as well as limited client report of improvement as compared to the second cluster. The therapists tended to use the ‘explicit exploration of clients’ implicit feelings’, ‘drawing interpersonal parallels,’ and ‘relational inquiries’ immediacy types. As such, it seems that in this cluster, immediacy was used primarily with the aim of fostering client insight regarding their way of relating in the context of good collaboration. The second, less common cluster, ‘mutual engagement in immediacy’, entails more complex immediacy processes characterised by conflict. More specifically, the clients' responses to immediacy conversations reflected both helpful (engagement in immediacy and reports of functionality improvement) and hindering processes (impairment in client‐therapist collaboration) to a larger extent than in the first cluster. The therapists employed a wide range of immediacy interventions, including the ‘explicit exploration of clients’ implicit feelings’ and ‘immediate repair,’ and to a lesser extent, ‘relational inquires’ and ‘drawing interpersonal parallels,’ presumably with the aim of balancing insight‐oriented and alliance‐oriented agendas. Since the coding of immediacy and the subsequent LCA model did not take into account the sequence between immediacy types and effects within IEs, the following interpretation of the LCA analysis does not claim causality or leading effect between the immediacy types and effects. Our interpretations are tentative and draw upon clinical theory and empirically‐based suggestions for the use of immediacy.

Based on the findings of this study, the majority of times that therapists initiated a conversation about the here‐and‐now, this was done in the context of good collaboration and was followed by relatively limited client engagement in a reflective exploration of the therapeutic relationship. This could be understood as evidence that, in most cases where immediacy was employed, the therapists' insight‐oriented interventions were associated with the client responding more or less collaboratively, with emotional exploration and relational insight.

Below, we present a brief extract that illustrates this process. The client was a young unemployed man, who had sought therapy due to depressive symptoms, increased hopelessness and suicidal ideation. The segment presented came from the fourth therapy session and the second of the total of six IEs that were identified in this session. The client had missed the previous session, and the possible meanings of this were discussed extensively throughout the session.

**Τ:** Yes, you have said that before, that (.) in a way eh, this has been happening lately and, as it's like a process that helps you feel better, not to think, as you say, to forget (.) [sighs] and I wonder if (.) all of this, in a way you feel that it is related to our early sessions (2)

**C:** Yes, like I thought this through more carefully

**T:** I don't know what you thought or didn't think about, I'm just wondering whether the things that, like, you would like to forget or, the need not to remember, like, to forget, to forget oneself, whether in a way it's connected to, the fact that, there were, there were three meetings and, anyway, quite a few things were said about the present, about the past (.) about you and

**C:** Yes maybe

**Τ:** as if the need arises in you to leave all this behind, you go out, you drink, you miss your previous, a session is missed due to the Bank holiday and then you miss your next session

**C:** Probably, yes, probably it is connected in a way like this, that all the issues that surfaced and (.) I wanted to forget them (.) I didn't want to face them (.) or I want to avoid them; and with our sessions (.) they may have become more intense and (5) it can be connected in this way, you're right (.)

**T:** So, I guess a part of you is reluctant to come (.) even though there is a part of you that eventually does, at least today

**C:** Yes, maybe a bit of me, a voice inside me, is reluctant (5) and there is generally a conflict inside me, Ok this is something I've known for a long time

In this extract, the therapist initiates an immediacy discussion focusing on the client's issue, which has been discussed in the previous IE ‐i.e. his need to forget, as a withdrawal stance, and she links to a defence. She attempts to explore the covert meaning of his absence and interprets it as his need to avoid painful thoughts and affects. After an initial hesitation, the client agrees with the therapist's interpretation and collaboratively engages in an immediacy discussion, contributing with meanings that enrich the construction of his defensive avoidance. The therapist formulates the concept of reluctance, the client takes this on and describes reluctance as an inner voice, part of an intrapsychic conflict. Following this IE, this conflict is further explored.

Concluding, it could be hypothesized that the evident association of a good therapeutic climate and the promotion of relational processing and insight with the therapists' invitations to relational processing in this cluster is in line with the view that the quality of the therapeutic alliance provides a facilitative context for the employment of strategic therapeutic agendas and the realization of specific therapeutic aims, such as transference work (Meissner 2007).

On the other hand, the features of the ‘mutual engagement in immediacy' cluster suggest complex immediacy processes. In about one‐third of the total immediacy events that belong in this cluster, clients were more likely to engage in immediacy discussions ‐ i.e., to express their feelings in the‐here‐and‐now and reflect upon them and to report a sense of improvement‐ in IEs characterized by a negative interpersonal climate. This may seem counter‐intuitive, given clients' engagement in immediacy has been found to be associated with effective and good‐quality immediacy events (Kuprian et al. 2022). One possible interpretation of this finding is that the impaired collaboration observed may have preceded the use of immediacy (e.g. client's nonattendance); in other words, the therapists may have used immediacy as a way of attending to and processing difficulties in collaboration, i.e., alliance ruptures or difficulties with engagement. Drawing upon the rupture resolution literature, addressing negative therapeutic processes by discussing what is happening in the here‐and‐now provides an opportunity to process client maladaptive interpersonal patterns and may help both to re‐establish collaboration and to foster client relational awareness, self‐reflection, and assertion in the‐here‐and‐now (Muran and Eubanks 2020; Safran and Muran 2000). In this study, these phenomena could be reflected in increased client engagement in immediacy. Another possible interpretation of the impaired therapeutic collaboration observed in this cluster may be that this reflects the often transient, negative immediate effect associated with the client's discomfort with the use of immediacy (Hill et al. 2014). Similarly, in the literature on alliance rupture resolution processes, it is suggested that sometimes the therapist's attempts to resolve ruptures may lead to tension, further rupture, and avoidance of exploration (Muran and Eubanks 2020).

With regard to immediacy types, in the ‘mutual engagement in immediacy’ cluster, therapists tended to use a range of different immediacy interventions, including both an insight‐oriented focus that aims to promote relational awareness and insight and a more supportive focus on maintaining the therapeutic alliance and promoting affect regulation, through empathy and validation of clients' feelings. This complex therapeutic agenda resembles descriptions of helpful therapeutic action during rupture repair processes as a complex flexible process entailing multiple therapeutic response options (e.g., Muran and Eubanks 2020). The therapists' skillful and responsive oscillation between the complementary and intersecting pathways of immediate and exploratory resolution paths is assumed to have beneficial effects on the therapy process, relatedness, and the client's exploratory engagement and metacommunication (Muran and Eubanks 2020).

All the above interpretations of ‘mutual engagement in immediacy’ cluster are illustrated by an extract taken from the same therapy presented above. The segment came from the 15th therapy session and from a quite long IE that occupied most of the session time. In this phase of treatment, the client's nonattendance had increased and before the extract presented below, the client had expressed his concern that his new long‐distance romantic relationship was likely to fail.

T: I have the sense though, that say, that your relationship here, with me, in your therapy is also at a distance (9)

C: Yes, but this is a completely different thing, the sessions are once a week anyway

T: Yes, but we do not meet every week (.)

C: A, once every 2 weeks (.)

T: I mean, (.) how long have we been meeting? Around 6 months, in therapy (.) It looks like (.) One would say, there are similarities, the same situation is created here too (.)

C: Yes, that's one point of view (.) Yes (.) From the point of view of frequency, perhaps, yes, ok

T: It is a therapy where our sessions are scheduled every week, but we meet at best every 2 weeks (.) While you pay for each week

C: Yes (.) Yes, but I just think that if I had the choice to meet with my girlfriend more often, I would love to, but if I had the choice for the sessions to be increased, I wouldn't, because I believe once a week is fine, but yes there is a similarity, we could say (18) I don't know, it doesn't mean anything to me this similarity (.) should it sound helpful to me? (.) I do not know how to think about it

Initially, the therapist provides a transference interpretation inviting the client to explore his relational issues. She focuses on the client's nonattendance and makes an explicit link between their interaction and his maladaptive interpersonal patterns. This interpretation is met by confusion and disagreement by the client, resembling a ruptured communication.

T: Look, I have the thought, that your romantic relationships are always, I have the sense that it's one thing you feel you need so much, you miss it, you need the contact, the other to be there for you, as you told me, let's say, to do the things I have to do and then to return, let's say to my “harbour”, but you often form long‐distance relationships (.) Treatment is something that, I don't think you come for fun

C: No

T: It's not an easy thing (.) E, I feel like it's hard to come and talk to me anyway, about things that you struggle with, so I want to say it's something that (.) you obviously want to do, you need to do, it's not a problem let's just say, okay (.) e, and it' s done in another, frequency, which, e, once a week is a, how can I say it is the lowest possible frequency for a therapy in which one expects changes, right? no (.) because you come, raising issues that are very serious, that concern your life, that concern (.) “What is happening to me, with my life, I see things happen, again and again”, that is, you don't come, okay, for something small, how to say it, I got stressed and now I came to see what is happening, so, you end up risking your life in terms of intention

C: Yes, ok

T: That's what I am saying, in that sense I do not know exactly what is happening, but I see (.)

C: That there is a similarity

T: Something is happening over here, hey?

C: Yes (26)

In the part of the interaction shown above, the therapist clarifies further her interpretation, she attends on the client's interpersonal needs of intimacy while she formulates his defensive attitude and his tendency to form long‐distance relationships. She also validates the client's defensive tendency to maintain distance in relationships, acknowledges his distress in therapy and invites him to a collaborative exploration of the here‐and‐now. The therapeutic dyad seems to be moving towards an increased alignment. After this part of interaction, the client continues to disagree with the proposed hidden meaning of his nonattendance and claims that practical reasons stop him coming to the sessions; it is worth noting that he adopts a collaborative and reflective manner. The therapist on the other hand, continues to invite the client to collaborative exploration.

C: I understand what you mean yes (12) I don't know what to say concerning the past, because (7) Okay I think that then the feeling of it being difficult getting here was stronger and that, I don't mean that it doesn't exist any more, that, it may still be the case, but certainly the conditions are much better than even two weeks ago or three or four

T: They are better, because you feel better

C: Yes

T: Therefore, when you feel stronger, then you want to come to therapy

C: Yes

T: But when you feel more

C: Weak

T: Weak, you don't want to, or you are reluctant

C: or I avoid it, yes

T: You avoid it

C: Yes

T: Because one would say that then the need is bigger?

C: Yes (5) Yes, and I don't believe that, I don't know in that particular phase, I didn't believe that being weaker, I would have more intense need, which I was feeling like that, someone would say, like you say, but for me it wasn't like this

T: How?

C: How it was?

T: Mmm

C: That to be exposed, makes me feel even more vulnerable and weak […]

This last extract illustrates a collaborative and explorative immediacy process. Eventually, the client reflects on the idea that he keeps his distance in therapy, while also having improved. The therapist tentatively constructs a new meaning for the client's and reluctance to come to therapy, as she connects it with his sense of vulnerability and fear of exposure. After this segment, the client continues to reflect and elaborate on his ambivalence and sense of exposure in therapy.

Τhe preliminary findings concerning the therapeutic strategy in the ‘mutual engagement in immediacy’ cluster align with the literature on effective transference work, which suggests that it is important to make use of multiple therapeutic actions and different mutative aspects of the therapeutic relationship (Gabbard and Westen 2003). Regarding the therapist technique, this literature suggests that a combination of transference interpretations alongside empathic, validating, and supportive interventions is most effective, as these strengthen the therapeutic alliance and create a holding environment for the client (Gabbard and Horowitz 2009).

Although preliminary, the findings of this study point to some implications regarding the use of immediacy as a way of processing relational dynamics in psychodynamic therapy. In this study, the therapeutic dyad's engagement in immediacy –which is considered evidence of effective use of immediacy– was associated with the presence of negative interpersonal dynamics, which could be conceptualized in terms of ruptures in the alliance. In other words, immediacy may be particularly helpful at times ‐both within sessions and over the course of treatment‐ where difficulties arise in therapeutic collaboration. Also, the findings of this study support the view that engaging clients in reflective immediacy discussions tends to be associated with the use, on the one hand, of interpretative interventions that focus on relational awareness or the transference and, on the other, tentativeness and empathic responsiveness (Hill et al. 2018; Muran and Eubanks 2020; Safran and Muran 2000). These findings support the view that the controversy that sometimes exists in psychodynamic clinical theory between the relative importance of insight, primarily through transference interpretations, versus the ‘holding environment’ (Winnicott 1965), which is associated with strengthening the therapeutic alliance (Gabbard and Westen 2003), is misleading.

### 4.1 Strengths, Limitations, and further Study

This study has several strengths. The observation of immediacy use by coders in different therapeutic dyads and the fact that the therapists were blind to the study's objectives provide a naturalistic description of immediacy use in a sample of psychodynamic therapies. In addition, to the best of our knowledge, this is the first study that examined the immediate effects of immediacy using empirically derived categories (Hill et al. 2018). Finally, the implementation of multivariate analysis Latent Class Analysis allowed us to systematically explore the association of immediacy types with different immediate effects, thus promoting our understanding of immediacy's role in the therapy process.

On the other hand, the small sample size of only seven therapies, with two therapists, limits the power of the LCA and the generalizability of the findings. This is compounded by the naturalistic design of the study, which has meant that the clients are not similar in terms of diagnosis and/or problem presentation. Additionally, there were no inter‐rater agreement scores for the IEs' coding due to our consensual strategy of coding immediacy, that does not rely on calculating reliability scores, rather than on taking into account multiple perspectives of the coding team during the in‐depth consensus discussions. The latter combined with the possible biased assessments of the coders, which is inherent risk in the Consensual Qualitative Approach consist limitations of the study and impose the careful consideration of results' interpretations. It is worth mentioning that some immediacy types and immediate effects occurred very rarely in our sample or not at all, and this is likely to have affected the power of statistical analysis. Concerning the statistical analysis, surprisingly, there were relatively few immediacy effects on the resulting model differentiating the whole sample of IEs into clusters. This fact has probably affected the power of analysis and our findings' interpretations, even though it was an analysis‐driven result. Furthermore, the fact that the LCA is an analysis of concurrent categories and not a time‐series analysis prevents us from assuming causality between immediacy types and effects and limits our ability to better understand their links, although preliminary provide interesting insights and hypotheses for further research.

Future research on the micro‐process of immediacy is needed to develop further models regarding links between immediacy types and immediate effects, with the broader aim to learn more about when and how to best use immediacy. Future studies could use mixed‐method with larger samples, coding immediacy on a session‐by‐session basis, and applying rigorous sequential time‐series analyses to further clarify the qualities of immediacy interventions and their impact on the client's responses. It would also be interesting to incorporate the therapists' and clients' characteristics and contextual factors as potential moderators of immediacy employment. Furthermore, an interesting future direction for the client's engagement in immediacy is its operationalization not just as an immediate effect but also through examining client‐initiated IEs.

Concluding, research on immediacy consists of a theoretically‐driven operationalization of relational processing as a strategic way of intervening, especially when problems arise in psychodynamic therapy. Its further investigation incorporating the therapeutic dyad's interpersonal dynamics is a promising research path for psychodynamic therapeutic action.

## Author Contributions

A.M. contributed to the study conception and design. Material preparation, coding was performed by A.M. and J.V. performed the statistical analyses. The first draft of the manuscript was written by A.M., E.A and J.V. All authors commented on previous versions of the manuscript, read and approved the final manuscript.

## Ethics Statement

The study was conducted in accordance with the Declaration of Helsinki and approved by the Ethics Committee of the Scientific Board of the Hellenic Centre of Mental Health and Research in September 2015.

## Consent

Informed consent was obtained from all subjects involved in the study. Consent for publication was obtained from all participants at the time of data collection.

## Conflicts of Interest

The authors declare no conflicts of interest.

## Data Availability

Data are available on request from thecorresponding author. The datafrom the video material are not publicly available due to ethical restrictions.
